# 

**Published:** 1878-11-20

**Authors:** 


ORIGINAL AND SELECTED ARTICLES.

A case of Post-Partum Hemorrhage with unusual complications—Recovery; by S. H. Anderson, M.D., of Missouri ....................321
Partial Paralysis and Incontinence of Urine, caused by Congenital Phimosis in a boy ten years of age; by B. M. Walker, M.D, of Virginia.......... ....................322
An ounce Minnie Ball imbedded in the Brain Fifteen Years successfully removed; by O. M. Doyle, M D., of Georgia ....................323
Evisceration without instruments; by J. T. Suggs, M.D., of Texas.......... ....................324
Society Reports—Medico Chirurgica Society—Reported by Dr. R. C. Word, 325, The use of the Hydrate of Chloral per rectum 327; Syphilis in the Negro. as differing from Syphilis in the White Race....................329
Diphtheria and its treatment; by H. A. Eberle. M D., C. M.......... ..........330
Pruritus Vulvæ treated with Suphurous Acid; by Ewd. B. Stevens, M.D, Lebanon, Ohio.331

ABSTRACTS AND GLEANINGS.

Stammering, 332. Hypodermic injection of Ergot in a case of Keloid: by Dr. J. E. Kempf, 333. Treatment of Lead Colic, 334. Cascara Sagrado in Constipation: by J. G. Sutton, M. D., Geneva, Ohio, 334. Translations—Cure of Onychia Maligna, 334. Spinal disease treated by Sayre's Plaster Jacket, 335 Genito-Urinary Surgery, 335. Oil of Amber in Anginose affections, 336. The influence of Salicylate of Sodium in the treatment of Diabetis, 336. To conceal the taste of Quinia, 336 Hiccough cured by Compression, 336. Valentine's Meat Juice, 337. Coto Bark, 337. Ailanthus Glandulosa, 337. Strychnia or Nux Vomica as a tonic, 338. Contraction of the fingers, 338. Treatment of Glandular Sore Throat, 339. Death from Ergot employed to cause Abortion, 339. Management of the Breasts where Nursing is not adopted, 339. Local Anaesthetic, 340. Hot Mustard Baths in Pneumonia of children, 340. Calomel, 340. Glycerole of Subacetate of Lead in Eczema; cases by Prof. Duhring, 341. On the use of Wine in the Capillary Bronchitis of children, 343. Neutral Acetate of Lead in Granular Conjunctivitis, 343. Treatment of Croup by injection of Liq. Ferri Sesquichlorid into the Larynx and Trachea, 343. The Colodion Bandage in the treatment of Umbilical Hernia, 344. Remarks on Extract of Malt and its combinations; by J. J. Mulheron, M.D., 344. Prognosis in Cerebral Hemorrhage, 346. Caffeine in Cephalalgia, 346.

NOTES, QUERIES AND FORMULÆ.

Bromide potassium in chronic chills. Vaseline in pharmacy. Alteration of calomel, 346. Eczema Capitis. Bronchial catarrh. Carbolic acid of ague, 348.

SCIENTIFIC ITEMS.

Illusions. Ballooning Spiders.

EDITORIAL AND MISCELLANEOUS.

Elegant display of chemicals. Practical journalism. Our Advertising department, 350. Taxing Medical men. National Yellow Fever Relief Commission. Rare chance. Copy of Dr. Redwood's report on Trommer's Extract of Malt. The Southern Presbyterian, 351. Book Notices.

